# Should Intercepting Traps Be Omitted?

**Published:** 1912-11-23

**Authors:** 


### HOSPITAL ARCHITECTURE AND CONSTRUCTION.

[Communications on this subject should be marked "Architecture" in the left-hand top corner of the envelope.]

### Should Intercepting Traps be Omitted?

Of all the details of construction probably none is less thought about by the average superintendent or architect than the intercepting trap to the drains where they enter a sewer. Yet this detail is sufficiently important to form subject for a seventy-seven page Report by a Departmental Committee to Parliament. Nominally, the investigation started with a proposal by the Willesden Urban Council to adopt a series of bye-laws for building control in which requirement as to the provision of an intercepting trap was to be omitted. The Local Government Board refused assent to this procedure, but promised the subject would be fully inquired into. In 1908 the Committee, which has now presented its Report,* was appointed. It consisted of an expert in each of the three subjects—architecture, civil engineering, and medicine, together with a secretary, and no less than fifty-two eminent witnesses of allied professions were examined.

The Report discusses at length the relative advantages and drawbacks of the intercepting trap, the chemistry and bacteriology of sewer air, the bacteriology of drain air, and the ventilation of sewers, together with general evidence and various experiments on animals.

The conclusions show that the intercepting trap has substantial disadvantages and drainage may be simplified and cheapened by its omission. It was found that 23 per cent. of 5,600 traps examined showed evidence of being blocked by solid matters, though, it is suggested, improvement in construction might remedy this. The function of the interceptor is by water-seal to prevent air from the sewer coming into the private drain and possibly into the buildings. It does fulfil this purpose, but experiments proved that animals were not affected in growth, nutrition, or susceptibility to disease by exposure to sewer air, though they might be adversely affected by exposure to the concentrated effects of putrefying excrementitious substances. Further epidemiological evidence confirms that human beings deliberately exposed to the effect of sewer air do not appear to be affected in health, and those districts where interceptors are not in vogue are not less healthy than others.

Sewer air is nevertheless objectionable on account of its smell. It is most important that public health authorities should make every effort to secure that sewers and drains are so designed and constructed that the opportunities of their contributing smell to the atmosphere shall be reduced in every possible way by making the tops of soil pipes and other ventilating shafts as remote as practicable from windows or ventilators and thereby also minimise the possibility of sewage microbes being wafted towards them.

* *Report of Departmental Committee on Intercepting Traps* (C.D. 6359). 1s. 1d. Wyman and Sons, Fetter Lane, E.C.

